# Supplementary material for: The genome sequence of the Barred Hook-tip, Watsonalla cultraria (Fabricius, 1775) (Lepidoptera: Drepanidae)
Source: Wellcome Open Res. 2025 Nov 5;10:618. [Version 1] doi: 10.12688/wellcomeopenres.25058.1 (PMC12770887; doi:10.12688/wellcomeopenres.25058.1)
Supplement: Peer review history

# Reviewer report: Reviewer response for version 1

**DOI:** 10.21956/wellcomeopenres.27624.r143619

**Published:** 2026-01-07

**Authors:** Charles Plessy

**Affiliations:** 1 Okinawa Institute of Science and Technology, Okinawa, Japan

The high quality of the data is supported by all the standard quality controls done in this report, which follows the standard Darwin Tree of Life template for genome data notes. The BlobToolKit plot is one of the cleanest ones I have seen, the assembly metrics are excellent, and the single-copy orthologue genes (BUSCO) are found at a high accuracy. I have compared the genome presented here to the one of *W. binaria* and the resulting pairwise plot displays a very sharp diagonal, which rules out major scaffolding errors.

 This said, I was disappointed that the sequenced specimen was a male, which means that the W chromosome was not sequenced for this species. I think that the article should 1) give a very brief introduction on the use of the ZW sex determination system in this clade, and 2) explain why a female was not sequenced.

 The heterozygocity is very low (0.06%) compared with what I am used to with other wild organisms. Can the authors comment on whether this is unexpected for this species as well?

Are sufficient details of methods and materials provided to allow replication by others?

Yes

Is the rationale for creating the dataset(s) clearly described?

Yes

Are the datasets clearly presented in a useable and accessible format?

Yes

Are the protocols appropriate and is the work technically sound?

Yes

Reviewer Expertise:

comparative genomics

I confirm that I have read this submission and believe that I have an appropriate level of expertise to confirm that it is of an acceptable scientific standard, however I have significant reservations, as outlined above.

# Reviewer report: Reviewer response for version 1

**DOI:** 10.21956/wellcomeopenres.27624.r138833

**Published:** 2026-01-05

**Authors:** Olli-Pekka Smolander

**Affiliations:** 1 University of Helsinki, Helsinki, Finland

The manuscript (data note) presents a high-quality, chromosomal-level genome assembly for *Watsonalla cultraria (*the Barred Hook-tip moth). The reported genome size is approximately 319 Mb with a scaffold N50 of 11.4 Mb. The genome has been assembled using a combination of PacBio HiFi long-read sequencing and Hi-C chromatin conformation capture. The assembly is highly contiguous, with 99.94% of the sequence assigned to 31 chromosomal pseudomolecules, including the Z sex chromosome. The mitochondrial genome has also been assembled and included in the assembly and as a separate database entry. Gene annotation has been done using Ensembl pipeline, with 16,011 protein-coding genes predicted.The mitochondrial assembly length (15.21 kb) aligns well with other recent Drepanidae assemblies, such as *Tethea ocularis* (15.28 kb) and *Tethea albicostata* (15.31 kb).

 The reported methodology is scientifically sound and consistent with the current best practices for reference genome assembly. The sampling strategy is clearly described, with adequate data provided for the collected specimens. The study used two different specimens for the PacBio and Hi-C sequencing steps which is understandable due to the size of the organism. Furthermore, it supported by the results that this is not a significant issue, looking at the the low observed heterozygosity (0.06%) and the high quality of the final scaffolding (suggesting the practical absence of chimeric sequences). The manuscript provides sufficient detail regarding the library preparation, sequencing platforms (PacBio Revio and HiSeq X Ten), and the bioinformatics pipeline ( *Hifiasm*, *purge_dups*, *YaHS, etc.*), ensuring reproducibility. The software versions and specific parameters are also appropriately documented.

 The data quality metrics presented are excellent and meet the standards required for reference-quality genomes. The assembly achieves a consensus Quality Value (QV) of 61.8, which significantly exceeds the Earth BioGenome Project benchmark of QV40, indicating a very high base-level accuracy. The completeness of the assembly is further supported by a BUSCO score of 98.7% (single = 98.5%, duplicated = 0.2%) against the lepidoptera_odb10 set, indicating that the vast majority of expected orthologs are present as single copies and intact. The Hi-C contact map validates the chromosomal scaffolding and the BlobToolKit analysis confirms the absence of significant contamination from non-target organisms. The assignment of 31 autosomes and the Z chromosome is consistent with expected karyotypes in Lepidoptera, and the identification of the Z chromosome in the male (ZZ) specimen is biologically appropriate. The assembly metrics (QV ~62, N50 11.4 Mb) compare favourably with other recent Lepidoptera assemblies produced by the consortium

 Overall, the manuscript provides a clear and concise contribution to the genomic resources available for the family Drepanidae.  All raw sequencing data and assembly accessions are clearly listed for the ENA. There are no major flaws in the study design, execution, or presentation of the data. Consequently, I have no criticisms. The article is scientifically sound in its current form.

Are sufficient details of methods and materials provided to allow replication by others?

Yes

Is the rationale for creating the dataset(s) clearly described?

Yes

Are the datasets clearly presented in a useable and accessible format?

Yes

Are the protocols appropriate and is the work technically sound?

Yes

Reviewer Expertise:

Multiomics, genomics, microbiome, bioinformatics, computational biology, ML/AI

I confirm that I have read this submission and believe that I have an appropriate level of expertise to confirm that it is of an acceptable scientific standard.

# Reviewer report: Reviewer response for version 1

**DOI:** 10.21956/wellcomeopenres.27624.r138828

**Published:** 2025-12-29

**Authors:** Ozren Polašek

**Affiliations:** 1 University of Split, Split, Croatia

The manuscript provides an account of sequencing of a moth species, contributing to wider understanding of biodiversity. The species determination is the single problem, as the angle of the phograph may be slightly miselading, as it does not provide a decent account of the tip. The applied sequencing methodology is appropriate and well-defined, thus leaving only a possible problem in speceis assignment.

Are sufficient details of methods and materials provided to allow replication by others?

Yes

Is the rationale for creating the dataset(s) clearly described?

Yes

Are the datasets clearly presented in a useable and accessible format?

Yes

Are the protocols appropriate and is the work technically sound?

Yes

Reviewer Expertise:

Hymenoptera taxonomy

I confirm that I have read this submission and believe that I have an appropriate level of expertise to confirm that it is of an acceptable scientific standard.
